# Llama-Derived Single Domain Antibodies Specific for *Abrus* Agglutinin

**DOI:** 10.3390/toxins3111405

**Published:** 2011-11-11

**Authors:** Ellen R. Goldman, George P. Anderson, Dan Zabetakis, Scott Walper, Jinny L. Liu, Rachael Bernstein, Alena Calm, James P. Carney, Thomas W. O’Brien, Jennifer L. Walker, Eric A. E. Garber

**Affiliations:** 1 Naval Research Laboratory, Center for Bio/Molecular Science and Engineering, 4555 Overlook Ave SW, Washington, DC 20375, USA; Email: george.anderson@nrl.navy.mil (G.P.A.); daniel.zabetakis@nrl.navy.mil (D.Z.); jinny.liu@nrl.navy.mil (J.L.L.); 2 National Research Council Postdoctoral Fellow resident at the Naval Research Laboratory, Center for Bio/Molecular Science and Engineering, 4555 Overlook Ave SW, Washington, DC 20375, USA; Email: scott.walper.ctr@nrl.navy.mil; 3 Nova Research Inc., 1900 Elkin Street, Suite 230, Alexandria, VA 22308, USA; Email: rachael267@gmail.com; 4 US Army-Edgewood Chemical Biological Center RDCB-DRB-C, 5183 Blackhawk Aberdeen Proving Ground, MD 21010, USA; Email: alena.calm@us.army.mil (A.C.); jpcarn@sandia.gov (J.P.C.); 5 Tetracore Inc., 9901 Belward Campus Drive Suite 300, Rockville, MD 20850, USA; Email: tobrien@tetracore.com (T.W.O.); jaldrich@tetracore.com (J.L.W.); 6 Food and Drug Administration, Center for Food Safety and Applied Nutrition, Office of Regulatory Science, Division of Bioanalytical Chemistry, 5100 Paint Branch Parkway, College Park, MD 20740, USA; Email: eric.garber@fda.hhs.gov

**Keywords:** abrin, single domain antibody, reversible refolding

## Abstract

Llama derived single domain antibodies (sdAb), the recombinantly expressed variable heavy domains from the unique heavy-chain only antibodies of camelids, were isolated from a library derived from llamas immunized with a commercial abrin toxoid preparation. Abrin is a potent toxin similar to ricin in structure, sequence and mechanism of action. The selected sdAb were evaluated for their ability to bind to commercial abrin as well as abrax (a recombinant abrin A-chain), purified abrin fractions, *Abrus* agglutinin (a protein related to abrin but with lower toxicity), ricin, and unrelated proteins. Isolated sdAb were also evaluated for their ability to refold after heat denaturation and ability to be used in sandwich assays as both capture and reporter elements. The best binders were specific for the *Abrus* agglutinin, showing minimal binding to purified abrin fractions or unrelated proteins. These binders had sub nM affinities and regained most of their secondary structure after heating to 95 °C. They functioned well in sandwich assays. Through gel analysis and the behavior of anti-abrin monoclonal antibodies, we determined that the commercial toxoid preparation used for the original immunizations contained a high percentage of *Abrus* agglutinin, explaining the selection of *Abrus* agglutinin binders. Used in conjunction with anti-abrin monoclonal and polyclonal antibodies, these reagents can fill a role to discriminate between the highly toxic abrin and the related, but much less toxic, *Abrus* agglutinin and distinguish between different crude preparations.

## 1. Introduction

Abrin, an extremely toxic protein isolated from *Abrus precatorius*, also known as the rosary pea plant, is a type 2 ribosomal inactivating protein (RIP). Type 2 RIPs, like abrin and ricin, are characterized by having two subunits: an A-chain that inhibits protein synthesis by catalyzing the deadenylation of 28S rRNA [1], and a lectin B-chain that facilitates the uptake of the toxin into the cell [2,3]. The A and B subunits are joined by a single disulfide bond. Abrin and ricin are very similar in both structure and mode of action [4]. They share 40% identity between their A chains, including the enzymatic active site and 60% homology between the B chains [5,6]. Although the lethality of both ricin and abrin depends on the route of administration and the species challenged [7], abrin has been reported to be more lethal than ricin [8,9]. Abrin has the potential to be used as a toxic adulterant or as a component in a weapon of mass destruction, making the development of methods to detect and neutralize it important.

Abrin exists as several variants that can be separated by chromatography into three fractions: abrin I, II, and III [10]. These fractions show differences in their toxicity and ability to inhibit protein synthesis [11], however, the variants have been determined to be immunologically indistinguishable [12]. Abrin fractions I and III have essentially identical A chains, but differing B chains [13]. The abrin commercially available for research is sold as a mixture of the abrin fractions I, II, and III. In addition to the abrin fractions, the rosary pea plant contains a less toxic component termed *Abrus* agglutinin which shares about 80% homology to abrin, but is several orders of magnitude less toxic [14]. Unlike abrin, the *Abrus* agglutinin consists of a tetramer of two A and two B subunits [14,15]. Notwithstanding these differences, most anti-abrin antibodies fail to distinguish between the abrin fractions and *Abrus* agglutinin proteins [12].

While conventional antibodies towards abrin, both polyclonal and monoclonal, have been utilized successfully in detection schemes [8,16,17,18,19,20,21], there is interest in the development of recombinant ligands. Both DNA aptamers and conventional antibody fragments (single chain antibodies; scFv) that bind abrin have been described [18,22]. The aptamers were able to detect abrin at concentrations as low as 1 nM (~64 ng/mL) in an assay using a molecular light switching reagent which changed luminescence when the aptamer bound target. Addition of BSA or ricin also caused changes in the luminescence when using several of the developed aptamers, indicating that specificity could be a problem with the reagents and assay format [22]. Human scFv specific for abrin were selected from a naïve scFv phage displayed library. Selected binders were converted to a Fab format and had affinities of ~50-100 nM enabling detection of abrin to 35 and 75 ng/mL with minimal cross-reactivity towards ricin [18].

Single domain antibodies (sdAb) are the recombinant variable heavy domains from the heavy chain only antibodies found in camelids and sharks [23,24]. Unlike conventional antibodies, and their recombinant binding domains such as scFv, many sdAb are able to refold and bind antigen after heat or chemical denaturation [25,26]. SdAb have been developed towards a wide variety of targets and in addition to their stability [27], they have been shown to have high affinity and specificity, equivalent to conventional antibodies and their derivatives [28,29,30].

In an effort to develop high affinity, specific, and thermal stable recognition reagents, we isolated abrin binding elements from immune libraries of llama-derived sdAb displayed on phage. We panned the library against a commercial abrin preparation as well as abrin fractions I, II, and III. Selected sdAb were characterized by their ability to bind abrin, its variants, and the *Abrus* agglutinin as well as their ability to refold after heat denaturation. The isolated sdAb with the best affinities were found to recognize commercial abrin and the *Abrus* agglutinin but not abrin fractions I, II, or III. We also isolated binders towards abrin fraction I. Herein we detail the evaluation and characterization of these binders.

## 2. Materials and Methods

### 2.1. Reagents

Commercial abrin, commercial abrin toxoid, and staphylococcal enterotoxin B (SEB) were purchased from Toxin Technology, Inc. (Sarasota, Fl). According to the product data sheet, the abrin toxoid had been prepared using a glutaraldehyde method. Abrin fractions I, II, and III as well as the abrin agglutinin were supplied by the FDA as previously reported [10]. Ricin, ricin A chain, ricin B chain, and *Ricinus communis* Agglutinin (RCA120) were from Vector (Burlingame, CA). Anti-abrin monoclonal antibodies (mAbs) 18E11 and 5F6 were provided by Tetracore, Inc. (Rockville, MD). Immunizations of two llamas were performed by Triple J Farms (Bellingham, WA). PhycoLink^®^ Streptavidin-R-Phycoerythrin PJ31S (SA-PE) was purchased from Prozyme (San Leandro, CA). Anti-histidine tag-Phycoerythrin was obtained from Columbia Biosciences Corp. (Columbia, MD). Phosphate buffered saline (PBS), Tween 20, and bovine serum albumin (BSA) were obtained from Sigma-Aldrich (St. Louis, MO)*.* The anti-M13 antibody was purchased from GE Healthcare (Piscataway, NJ). Enzymes for PCR and cloning were obtained from Invitrogen Corp. (Carlsbad, Ca) and New England Biolabs (Ipswich, MA).

### 2.2. Abrax

Abrax is an abrin A chain sequence recombinantly expressed in *Escherichia coli* which has been modified to include the mutations described for the ricin A chain vaccine [31]. Abrax was expressed from a pET28a in *E. coli* using autoinduction media. A single colony from the overnight growth was used to inoculate 10 mL of Overnight Express Instant TB Autoinduction media (Novagen) with 30 μg/mL kanamycin. The culture was grown to log phase in a shaking incubator at 37 °C. The culture was then scaled up to one liter and incubated for 20 h at 37 °C in a shaking incubator. The cell paste was harvested by centrifugation and frozen. The cell pellet was thawed and resuspended in 30 mL of His Tag binding buffer (20 mM Sodium Phosphate, 500 mM NaCl, 20 mM Imidazole, pH 8) to which 300 μL of HALT EDTA free protease inhibitor cocktail (Fisher Scientific PI78415) was added. The cells were mechanically ruptured by two passages of the slurry through a French Pressure Cell Press at 40,000 PSI. The crude extract was clarified by centrifugation at 100,00× *g* and the soluble phase was loaded onto a pre-packed 1 mL Ni Sepharose column (GE Life Sciences) and abrax was step eluted into 20 mM Sodium Phosphate, 500 mM NaCl, 500 mM Imidazole, pH 8. Peak Fractions were pooled and buffer exchanged into PBS.

### 2.3. Library Construction and Selection

Two llamas underwent a series of 9 immunizations with commercial abrin toxoid; the amount of antigen per immunization was increased from 10 μg on days 0 and 7 up to 100 μg per injection on days 42, 56, and 84 of the protocol. On day 98, blood was collected from the immunized animals. 

White blood cells were isolated from 100 mL of llama blood and RNA was isolated using the QIAamp blood mini kit (Qiagen, Valencia, CA). RNA was used in an oligo-dT primed reverse transcription reaction. PCR amplification of the heavy domain antibodies was performed using flanking primers as described previously [32,33]. The resulting sdAb genes were digested, gel purified, and cloned into phage display vector pecan21 [33,34]. 

Selections for abrin binders were carried out essentially as previously described [33]. During the course of this work, the library was panned against the commercial abrin, abrax, and purified abrin fractions. The only alteration to the previously published panning protocol was that during the third round selections, wash buffer was left in the wells for 30 min between the PBST (PBS with 0.05% Tween-20) and PBS wash sets in an effort to eliminate lower affinity binders. 

Monoclonal phage enzyme linked immunosorbent assays (ELISAs) were performed after the first, second and third rounds of panning to identify individual positive clones. Luminex was used to confirm positive clones [35]. Selected positive clones were sequenced to identify unique sdAb genes, and sequences were aligned using the Multalin program [36]. Representative sdAb clones were subcloned from the phage display sdAb-fusion vector to a soluble sdAb expression vector, and transformed into *E. coli* Rosetta (Novagen, Madison, WI) for protein production as described previously [33,37].

### 2.4. Luminex Assays

Luminex (Austin, TX) carboxylated microspheres were cross-linked to a variety of proteins using the two-step carbodiimide coupling protocol provided by the manufacturer. Direct binding and sandwich assays were performed as previously described [37,38,39]. The signal for Luminex experiments is reported as the median fluorescence intensity (MFI) of at least 100 separate microspheres.

### 2.5. Surface Plasmon Resonance (SPR)

The SPR kinetic measurements were performed using the ProteON XPR36 (Bio-Rad Laboratories, Hercules, CA). GLC chips coated with the various toxins (commercial abrin, abrax, *Abrus* agglutinin, and abrin fractions I, II, and III), were used to measure the kinetics of anti-abrin sdAb binding. The toxins were immobilized to the chips, each at 5 µg/mL in acetate buffer (pH 5.0), according to the standard EDC coupling chemistry provided by the manufacturer. Association and dissociation between the sdAb (injected at six concentrations ranging from 0 to 30 nM) and the immobilized toxins were monitored for 180 and 600 s, respectively, using a flow rate of 50 µL/min. When evaluating mAbs, concentrations ranging from 0 to 30 nM were used. The chip was regenerated using 50 mM glycince-HCl (pH 2.0) for 36 s, prior to any additional testing. All experiments were performed at 25 °C. The data were analyzed using the ProteON Manager^TM^ 2.1 software. The binding constants were determined using the software’s Langmuir model after subtracting the zero antibody concentration column and interspot correction. A bivalent fit was used to analyze the data from the mAbs.

### 2.6. Circular Dichroism (CD)

The melting points of selected sdAb were measured by circular dichroism using a Jasco J-815 CD spectropolarimeter equipped with a PTC-423S single position peltier temperature control system. Samples (~25 µg/mL) were prepared by dialysis *versus* 5 mM sodium borate pH 8.0, or by dilution. Measurements were made in a 10 mm pathlength quartz cuvette with a stir bar. Melting point data were acquired at a single wavelength between 202 and 210 nm, at a temperature rate of 5 °C/min over the range of 25 °C to 95 °C.

## 3. Results and Discussion

A library of phage displaying sdAb, derived from two llamas immunized with a commercial abrin toxoid, was constructed. After performing two rounds of selection using commercial abrin, 48 clones each from the first and second rounds of panning were evaluated by monoclonal phage ELISA to evaluate their ability to bind to the commercial abrin preparations. About 55% of the clones selected after the first round of panning and 70% of the clones selected after the second round were positive with a signal-to-noise ratio of at least 3. Forty of the binding phages from the first and second rounds, along with three non-binders, were examined by Luminex to assess the affinity and specificity to the commercial abrin, abrax (recombinant abrin A-chain), ricin, ricin-A chain, and ricin B-chain (Figure S1). Only one phage showed binding to the abrax coated microspheres. Of the positives, 23 that were strongly positive by both ELISA and Luminex were sent for DNA sequencing. Sequences fit into six unique families (typified by Abr1, Abr2, Abr4, Abr5, Abr6, and Abr11; Figure S2). Several clones that were positive by ELISA did not show appreciable signal when tested by Luminex phage direct binding assays and were not sequenced or evaluated further.

**Figure 1:**
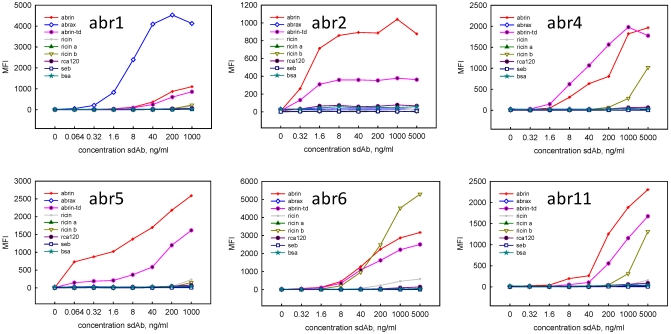
Luminex direct binding, representatives of the first six sequence families isolated through panning two rounds on commercial abrin. The sdAb were examined for binding to commercial abrin (abrin), abrax, and commercial abrin toxoid (abrin-td) as well as the related toxin ricin, ricin a chain, ricin b chain, and RCA120, and unrelated proteins staphylococcal enterotoxin B (SEB), and bovine serum albumin (BSA).

Representatives from each of the six unique sequence families were mobilized to the protein expression vector and soluble protein was produced. Direct binding experiments were performed using the Luminex to determine the specificity of the clones (Figure 1). Clones Abr1, Abr2 and Abr 5 appeared to be the most specific for commercial abrin and commercial abrin toxoid, showing no binding to ricin, the ricin A or B- chains, RCA120, or unrelated proteins SEB and BSA. The Abr 1 clone showed the highest signal with abrax, and was the only one of the six binding families that recognized the abrax. Clones Abr4 and Abr11 displayed moderate cross-reactivity with ricin B chain at high sdAb concentrations, while Abr6 showed strong cross-reactivity to ricin B chain. 

Only one abrax binding family was identified containing only one member, Abr1. To isolate additional sdAb that recognized abrax, two rounds of selection were performed using the abrax target. All 32 binding sequences from these selections fell into the same sequence family as Abr1. These results suggest that there are not many members of the library with high affinity for the abrin A chain or that other A chain binders make important contacts with the amino acids that have been changed in the abrax construct. Camelid sdAb have been shown to bind to enzyme active sites [40], accessing epitopes that are not available to conventional antibodies. 

**Figure 2:**
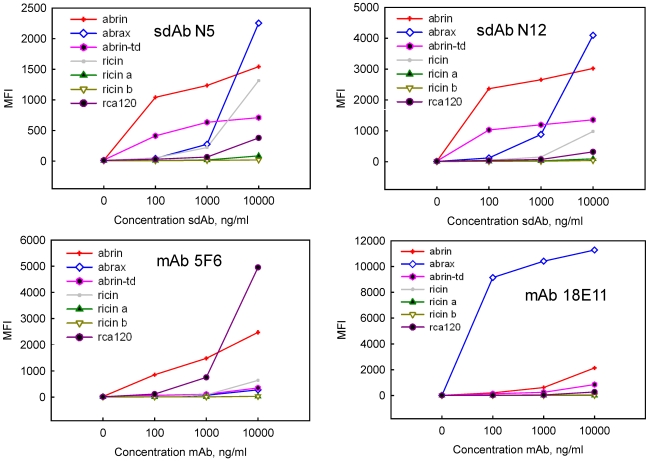
Luminex direct binding, representatives of the two sequence families isolated after panning three rounds on commercial abrin and two anti-abrin mAbs. The sdAb and mAbs were examined for binding to commercial abrin (abrin), abrax, and commercial abrin toxoid (abrin-td) as well as the related toxin ricin, ricin a chain, ricin b chain, and rca120. Note the difference in the scale of the Y axis between the mAbs and sdAb.

A third round of selection on the commercial abrin employed a longer washing steps in an effort to further down select binders with high affinity; two additional sequence families, N5 and N12 were identified (Figure S2). These binders were evaluated by Luminex direct binding assays, along with two anti-abrin monoclonal antibodies (mAbs), to determine their specificity. Clone N12 showed high specificity while N5 cross-reacted with ricin at the highest concentration examined (Figure 2). Sandwich assays demonstrated that the Abr 2 and N5 bound to different epitopes on the toxin (Figure S3) and could potentially be used as a captor tracer pair.

**Figure 3:**
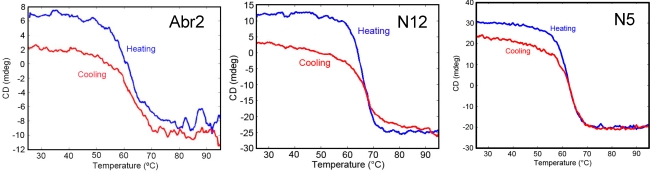
Circular dichroism on the three sdAb that showed high affinity and specificity to the commercial abrin toxin. Each sdAb was monitored through one heating and cooling cycle.

One advantage of sdAb over conventional antibodies is their ability to refold after heat denaturation. Circular dichroism analysis of Abr2, N5, and N12 confirmed that all three clones regained at least 75% of their secondary structure after heating to 95 °C (Figure 3). While most sdAb examined are able to refold and to bind antigen after denaturation, it is important to determine the ability of each clone to refold as some sdAb do not regain structure and function after heating [41].

The association and dissociation rate constants for commercial abrin, abrax, abrin fraction I, and *Abrus* agglutinin are listed in Table 1 and Table 2 (representative SPR data in Figure S4). The sdAb that had been identified as the best binders, Abr2, N5, and N12 were found to have sub nM affinities toward commercial abrin, the antigen that they were selected against, and *Abrus* agglutinin. However, Abr2, N5, and N12 did not bind abrax or the purified abrin fractions (data for abrin fractions II and III not shown). Abr4 and Abr6, which displayed cross-reactivity with ricin B-chain, did not bind abrax but bound commercial abrin, *Abrus* agglutinin, and all three abrin fractions with greatest affinity for abrin fraction I (data not shown for fractions II and III). Although the three abrin fractions had been reported to be immunologically indistinguishable [12], there are reports of anti-ricin antibodies that react with only one of the analogous 3 ricin fractions [42]. Thus, the preferential recognition of fraction I by Abr4 and Abr6 may represent inclusion in the epitope of sequence differences between the fractions that were not observed previously. 

**Table 1:** Binding to commercial abrin and abrax.

Binder	Commercial Abrin			Abrax		
	on, 1/Ms	off, 1/s	Kd, M ^a^	on, 1/Ms	off, 1/s	Kd, M ^a^
mAb 5F6	1.5 × 10^5^	6.1 × 10^−4^	4.0 × 10^−9^	NB	NB	NB
mAb 18E11	2.0 × 10^5^	1.9 × 10^−2^	9.3 × 10^−8^	1.5 × 10^5^	3.4 × 10^2^	2.2 × 10^−7^
Abr1	NB	NB	NB	1.8 × 10^5^	4.79 × 10^5^	2.64 × 10^−10^
Abr2	2.2 × 10^6^	3.2 × 10^−5^	1.5 × 10^−11^	NB	NB	NB
Abr4	4.2 × 10^5^	2.7 × 10^−4^	6.5 × 10^−10^	NB	NB	NB
N5	1.6 × 10^5^	3.1 × 10^−5^	1.9 × 10^−10^	NB	NB	NB
N12	3.7 × 10^6^	4.5 × 10^−6^	1.2 × 10^−12^	NB	NB	NB
Abr6	8.3 × 10^4^	1.1 × 10^−4^	1.3 × 10^−9^	NB	NB	NB
3E	3.3 × 10^5^	2.4 × 10^−4^	7.4 × 10^−10^	NB	NB	NB
10C	8.6 × 10^5^	9.7 × 10^−4^	1.1 × 10^−9^	NB	NB	NB

NB = no binding observed; ^a^ Kd values are calculated from kd/ka; Binding constants for mAbs were determined using a bivalent model while those for sdAb were done with a Langmuir model.

**Table 2:** Binding to abrin fraction I and abrin agglutinin.

Binder	Abrin frac I			*Abrus* Agglutinin		
	on, 1/Ms	off, 1/s	Kd, M ^a^	on, 1/Ms	off, 1/s	Kd, M ^a^
mAb 5F6	1.6 × 10^5^	12.1 × 10^−4^	1.3 × 10^−9^	1.5 × 10^5^	6.3 × 10^−4^	4.3 × 10^−9^
mAb 18E11	1.2 × 10^5^	1.1 × 10^−2^	8.5 × 10^−8^	1.8 × 10^5^	1.6 × 10^−2^	9.0 × 10^−8^
Abr1	NB	NB	NB	NB	NB	NB
Abr2	NB	NB	NB	1.1 × 10^6^	1.6 × 10^−5^	1.4 × 10^−11^
Abr4	1.5 × 10^5^	1.6 × 10^−4^	1.1 × 10^−9^	5.1 × 10^5^	4.9 × 10^−4^	9.5 × 10^−10^
N5	NB	NB	NB	1.8 × 10^5^	5.3 × 10^−5^	2.9 × 10^−10^
N12	NB	NB	NB	1.6 × 10^6^	4.2 × 10^−6^	2.6 × 10^−12^
Abr6	4.0 × 10^4^	2.1 × 10^−4^	5.2 × 10^−9^	9.1 × 10^4^	2.6 × 10^−5^	2.9 × 10^−10^
3E	1.2 × 10^5^	1.6 × 10^−4^	1.3 × 10^−9^	5.3 × 10^5^	4.2 × 10^−4^	7.8 × 10^−10^
10C	3.2 × 10^5^	6.5 × 10^−4^	2.0 × 10^−9^	1.2 × 10^6^	8.1 × 10^−4^	6.6 × 10^−10^

NB = no binding observed; ^a^ Kd values are calculated from kd/ka; Binding constants for mAbs were determined using a bivalent model while those for sdAb were done with a Langmuir model.

While SPR experiments showed Abr1 to have a high affinity for abrax, Abr1 did not bind commercial abrin, abrin fraction I, or *Abrus* agglutinin, indicating that it may recognize an epitope that is partially in the interface of the A and B chains and only accessible when the abrin A chain is separate. Thus, sdAb Abr1 may be useful to discriminate purified abrin A chain from the whole toxin. By itself the abrin’s A chain is not a toxic threat, but there may be circumstances in which it is important to differentiate the whole toxin from its component chains, especially when engineered immuno-toxins are real possibilities. 

**Figure 4:**
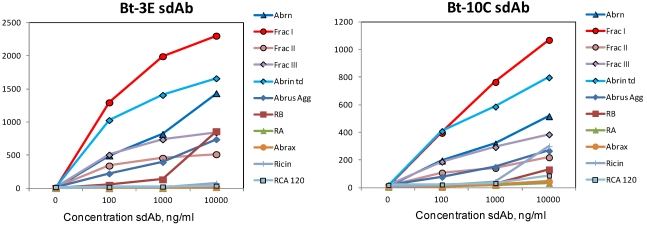
Luminex direct binding, representatives of two clones isolated after panning on the abrin fractions. Binding data is shown for commercial abrin (abrin), the abrin fractions (Frac I, Frac II, and Frac III), the commercial abrin toxoid (abrin td), the *Abrus* agglutinin (Abrus Agg), ricin b chain (RB), ricin a chain (RA), Abrax, ricin, and RCA120.

Having ascertained that initially selected sdAb bound best to either the agglutinin or to abrax, selections of the constructed phage library were carried out on the abrin fractions in the hope of isolating high affinity sdAb specific for the toxin. Two rounds of selection were performed and 24 clones that were positive by monoclonal phage ELISA were sent for sequencing; all isolated binders were from the existing families of Abr1, Abr4 and Abr6. Two binders, 3E and 10C, in the Abr4 family were chosen for further evaluation and shown to bind to the abrin I fraction (Figure 4; Table 2). These representatives would be expected to perform equivalent to Abr4 in sandwich assays, which had not shown particularly promising results.

In addition to the anti-abrin sdAb, two anti-abrin mAbs were evaluated. Tight binding was observed between mAb 5F6 and commercial abrin (Kd of 4 nM) while mAb 18E11 displayed poor binding (Kd of 93 nM). In contrast, no binding was observed between mAb 5F6 and abrax while mAb 18E11 displayed a Kd of 0.2 μM. Both mAb 5F6 and mAb 18E11 bound fraction I and *Abrus* agglutinin with Kd values <0.1 μM. When comparing these values to the affinity constants of the sdAb, it is important to realize that a bivalent model was used to calculate binding constants for the mAbs. As each mAb has two antigen binding sites, its effective binding to the abrin benefits from an avidity component, that increases its apparent affinity.

**Figure 5:**
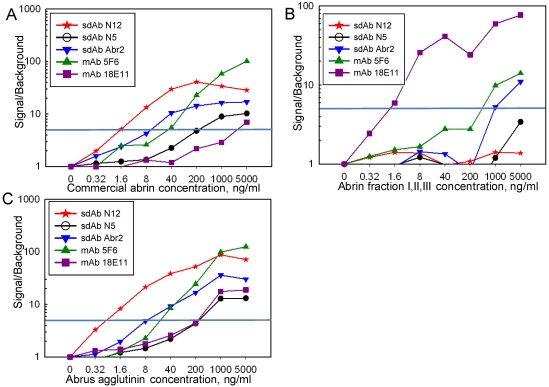
Sandwich assay using the Luminex for commercial abrin (panel A), abrin fractions I II and III (panel B), and *Abrus* agglutinin (panel C) with a sdAb and monoclonal captures paired with a llama polyclonal tracer. Data is reported as the ratio of signal over background, with a value of 5 considered positive.

Sandwich assays were performed on the Luminex instrument for the detection of commercial abrin, abrin fractions and the *Abrus* agglutinin using the sdAb, mAbs, and polyclonal llama anti-abrin (Figure 5). The data were plotted as the ratio of the signal at a given abrin concentration to that of the signal when no abrin was added. A ratio of at least 5 was chosen to indicate a positive response. Commercial abrin was readily detected using sdAb as either the capture or tracer antibody. The best limit of detection, 1.6 ng/mL, was achieved when the N12 sdAb was paired with a llama anti-abrin polyclonal as the tracer antibody. When mAb 18E11 was used as a capture reagent in combination with the llama anti-abrin polyclonal tracer, a comparable LOD value of 1.6 ng/mL was achieved with a mixture of abrin fractions I, II and III. Although the Abr4 family of sdAb has a good affinity towards the purified abrin fractions according to the SPR measurements, none of the sdAb were able to detect the abrin fractions when used as a tracer in combination with the mAbs, sdAb N12, or llama anti-abrin capture (Figure S5). The inability of the Abr4 family sdAb to function well in sandwich assays may be due to the presence of reactive amines at or near the combining site for this family of sdAb. Since the addition of biotin is amine directed, it would then have the potential to interfere with binding. The Abr4 family has multiple Arginines within the hyper variable regions (2 within complementarity determining region (CDR) 2, and 2 within CDR 3), and several in framework regions that are not present in the other families of abrin-binding sdAb; biotinylation at these sites could potentially interfere with abrin binding. It is interesting to note that the Abr4 family sdAb were not able to function as capture reagents in Luminex assays; since the EDC coupling employed is also amine directed, they may immobilize in an orientation that does not permit target binding. The N12 sdAb capture and the Abr2, N5, and N12 sdAb tracers, however, were useful in the detection of the agglutinin, with the best pairs showing signals at concentrations as low as 1.6 ng/mL (Figure 5; Figure S5). 

The material used for the original immunizations was the commercial abrin toxoid, a toxoid version of commercial abrin. Commercial abrin is a mixture of fractions I, II, and III, and *Abrus* agglutinin. Native and denaturing gels of the commercial abrin displayed bands almost identical to the *Abrus* agglutinin but considerably different from the purified fractions (Figure S6), indicating that *Abrus* agglutinin is present at a considerably greater concentration than the toxin variants. Therefore, it was not surprising that *Abrus* agglutinin binders were isolated. However, the lack of sdAb specific for abrin was a disappointment. Possibly the phage display repertoire of sdAb were dominated by the most immunogenic component present in the immunization, reiterating the importance of antigen purity [43]. Nonetheless, in combination with an antibody that recognizes abrin, such as the llama polyclonal anti-abrin, an assay that discriminates between abrin and the *Abrus* agglutinin can be developed using the sdAb and mAbs. Such an assay enables different sources and preparations of abrin to be distinguished and can serve as a key component in determining attribution in criminal cases.

## 4. Summary

Specific and high affinity sdAb recognition elements for *Abrus* agglutinin have been isolated. By combining these sdAb with either llama polyclonal anti-abrin antibodies or commercially available mAbs specific for abrin, it is possible to quantitatively determine the abrin and *Abrus* agglutinin composition of samples. Since commercial and crude home preparations routinely contain *Abrus* agglutinin, the material’s composition may serve as a fingerprint for attribution and threat agent forensics. Isolation of high affinity sdAb specific for abrin fractions I, II, and III, remains an important goal that may require sdAb libraries developed after immunization with either toxoids derived from the purified abrin fractions or active toxins/subunits. In addition to detection, a goal of sdAb development has been to isolate reagents able to inhibit toxin activity. SdAb have been described that recognized ricin A chain and inhibit ricin activity in cell culture [38]. There have also been reports of sdAb that neutralize botulinum neurotoxins [44,45,46,47]. Recently an abrin A chain binding monoclonal antibody was shown to completely inhibit abrin activity both in cell culture and in mice [48], showing that antibodies can be isolated that inhibit abrin activity.

## Supplementary Files

Supplementary File 1: PDF-Document (PDF, 916 KB)
