# Supplementary material for: Llama-Derived Single Domain Antibodies Specific for Abrus Agglutinin
Source: Toxins (Basel). 2011 Nov 11;3(11):1405–19. doi: 10.3390/toxins3111405 (PMC3237003; doi:10.3390/toxins3111405)
Supplement: Supplementary File 1: — PDF-Document (PDF, 916 KB) [file toxins-03-01405-s001.pdf]

Supplementary Information

Ellen R. Goldman [1,]*, George P. Anderson [1], Dan Zabetakis [1], Scott Walper [2], Jinny L. Liu [1], Rachael Bernstein [3], Alena Calm [4], James P. Carney [4], Thomas W. O'Brien [5], Jennifer L. Walker [5] and Eric A.E. Garber [6]

[1] Naval Research Laboratory, Center for Bio/Molecular Science and Engineering, 4555 Overlook Ave SW, Washington, DC 20375, USA; E-Mails: george.anderson@nrl.navy.mil (G.P.A.); daniel.zabetakis@nrl.navy.mil (D.Z.); jinny.liu@nrl.navy.mil (J.L.L.)

[2] National Research Council Postdoctoral Fellow resident at the Naval Research Laboratory, Center for Bio/Molecular Science and Engineering, 4555 Overlook Ave SW, Washington, DC 20375, USA; E-Mail: scott.walper.ctr@nrl.navy.mil

[3] Nova Research Inc, 1900 Elkin Street, Suite 230, Alexandria, VA 22308, USA; E-Mail: rachael267@gmail.com

[4] US Army-Edgewood Chemical Biological Center RDCB-DRB-C, 5183 Blackhawk Aberdeen Proving Ground, MD 21010, USA; E-Mails: alena.calm@us.army.mil (A.C.); jpcarn@sandia.gov (J.P.C.)

[5] Tetracore Inc., 9901 Belward Campus Drive Suite 300, Rockville, MD 20850, USA; E-Mails: tobrien@tetracore.com (T.W.O.); jaldrich@tetracore.com (J.L.W.)

[6] Food and Drug Administration, Center for Food Safety and Applied Nutrition, Office of Regulatory Science, Division of Bioanalytical Chemistry, 5100 Paint Branch Parkway, College Park, MD 20740, USA; E-Mail: eric.garber@fda.hhs.gov

* Author to whom correspondence should be addressed; E-Mail: ellen.goldman@nrl.navy.mil; Tel.: +1-202-404-6052; Fax: +1-202-767-9594.

Received: 2 September 2011; in revised form: 23 October 2011 / Accepted: 1 November 2011 / Published: 11 November 2011

Abstract: Llama derived single domain antibodies (sdAb), the recombinantly expressed variable heavy domains from the unique heavy-chain only antibodies of camelids, were isolated from a library derived from llamas immunized with a commercial abrin toxoid preparation. Abrin is a potent toxin similar to ricin in structure, sequence and mechanism of action. The selected sdAb were evaluated for their ability to bind to commercial abrin as well as abrax (a recombinant abrin A-chain), purified abrin fractions, *Abrus* agglutinin (a protein related to abrin but with lower toxicity), ricin, and unrelated proteins. Isolated sdAb were also evaluated for their ability to refold after heat denaturation and ability to be used in sandwich assays as both capture and reporter elements. The best binders were specific for the *Abrus* agglutinin, showing minimal binding to purified abrin fractions or unrelated proteins. These binders had sub nM affinities and regained most of their secondary structure after

heating to 95 °C. They functioned well in sandwich assays. Through gel analysis and the behavior of anti-abrin monoclonal antibodies, we determined that the commercial toxoid preparation used for the original immunizations contained a high percentage of *Abrus* agglutinin, explaining the selection of *Abrus* agglutinin binders. Used in conjunction with anti-abrin monoclonal and polyclonal antibodies, these reagents can fill a role to discriminate between the highly toxic abrin and the related, but much less toxic, *Abrus* agglutinin and distinguish between different crude preparations.

Keywords: abrin; single domain antibody; reversible refolding

---

Figure S1. Luminex direct binding of llama anti ricin (L < R) and llama anti abrin (L < A) polyclonal antibodies as well as phage displayed sdAb. Binding examined for commercial abrin (Abrin), abrax (A ac), ricin, ricin a chain (R ac) and ricin B chain (R bc). Clones 1B, H11, and G12 were negative by monoclonal phage ELISA and are also negative by the Luminex direct binding assay.

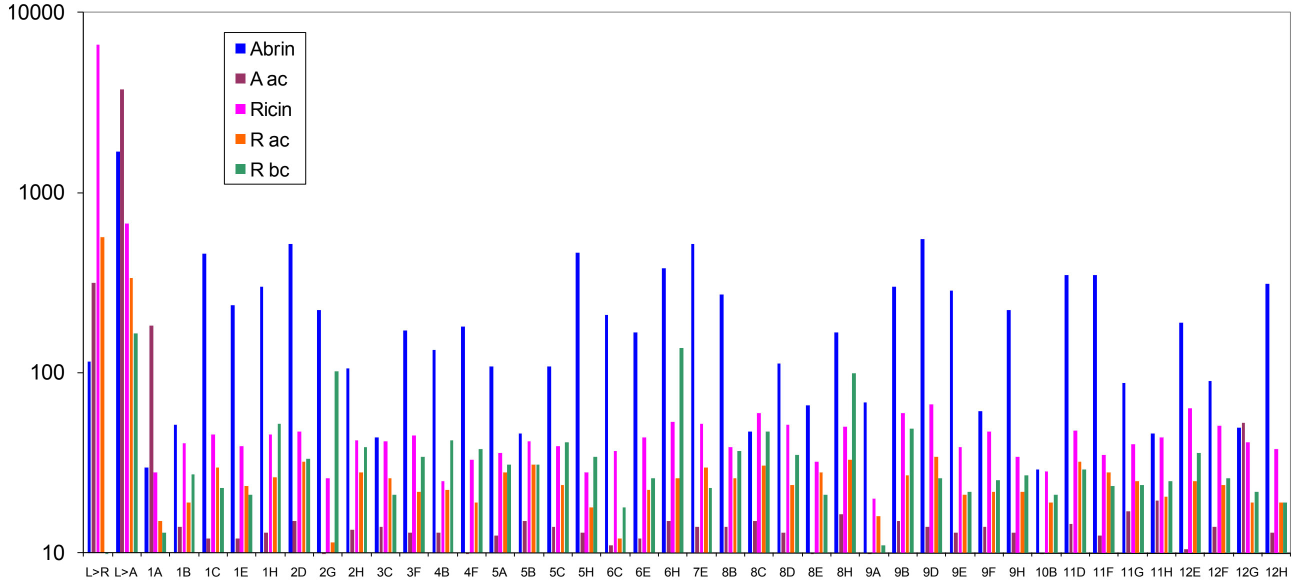

Figure S2. Amino acid sequence determined from DNA sequencing of the unique anti-abrin sdAb characterized for this work. These 10 clones represent 8 sequence families.

```
       1        10        20        30        40        50        60        70        80        90        100       110       120 124
       |--------+---------+---------+---------+---------+---------+---------+---------+---------+---------+---------+---------+---|
 ABR1  EVQLQASGGGLVQAGGSLRLSCAASGSDGSLEIMGWYRQAPGKQRELVARATSGGLK-NYADSVKGRFTISGDLDKNTVYLQMNSLQPEDTGVYYCHLGVVA-STYR----DYWGQGTQVTVSS
 ABR6  EVQLQASGGGLVQAGGSLRLSCVVSGTTISISAVGWYRQAPGKERDMVARISSGGSNTDYVANVQGRFTVSRDNAKNTAYLQMNNLKPEDTAVYYCHVRPATWGQSR----GYWGQGTQVTVSS
 ABR2  DVQLQASGGGLVQAGGSLVLSCTASGRSVSSLAMGWLRQVPGKERELVGSISLSGGRTYYADSMKGRFTISKDNAKNTVALQMNSLRPEDTAVYYCAARDAARDYAPLN-FDTWGQGTQVTVSS
  N12  EVQLQASGGGMVQGGGSLRLSCAASAHTFSDYAMGWFRQAPGKERSLVAAINRSGGRTWYTDSVRGRFTISRDNAKNTVYLQMDKLKPEDTAVYYCAAKSTHRDYAALNDYNYWGQGTQVTVSS
   N5  EVQLEESGGGLVQPGGSLRVSCAASGFAFSGYAMGWFRQAPGKEREFVAGISRTDNYRPYADSVKGRFAISRDNAQNMVYLQMNSLKPEDTAVYYCAADRLGVPTPNSADYQYWGQGTQVIVSS
 ABR4  EVQLVESGGGLVQAGRSLILSCSASGGIFTNMGMGWYRQAPGKQREVVAQITNRG-RTNYGESVKGRFTISRDNAQRTAYLQMNSLKPEDTAVYYCRTNYGGR--------DYWGQGTQVTVSS
   3E  EVQLVESGGGLVQAGRSLILSCSASGGIFANMGMGWYRQAPGKQREVVAQITNRG-RTNYGESVKGRFTISRDNAQRMAYLQMNSLKPEDTAVYYCRTNYGGR--------DYWGQGTQVTVSS
  10C  EVQLVESGGGLVQAGRSLILSCSASGGIFTNMGMGWYRQAPGKQREVVAQITNRG-RTNYGESVKGRFTISRDNAQRMAYLQMNSLKPEDTAVYYCRTNYGGR--------GYWGQGTQVTVSS
 ABR5  EVQLQASGGGLVQAGGSLRLSCVVSGLSVGLNPVAWYRQAPGQQRDWVARMSMRG-LTDYPDSLKGRAIISRDNAKNTVSLQMDSLKPEDTAVYFCKVGL-----------SYWGQGTQVTVSS
ABR11  DVQLQASGGGLVRAGGSLRLSCAVSGLTVSHAPSAWYRQAPGKQRDWVARLSMRG-LTDYEDSVKGRFTISRDNAKNTVYLQMDSLKPEDTAVYFCKAGL-----------NYWGQGIQVTVSS
```

Figure S3. Sandwich assays showed the Abr 2 and N12 bound to the same epitope on the *Abrus* agglutinin, but N5 bound to a different epitope than N12. Left panel: A ProteON GLC chip coated with *Abrus* agglutinin was exposed to the sdAb N12 at 30 nM in lanes 2–5 (light blue, dark blue, green and pink), lane 1 (red) and 6 (orange) were buffer blanks. Right panel: To evaluate epitope location the same area was challenged with Abr 2 30 nM (lanes 1–3) and N5 30 nM (lanes 4–6). Abr 2 generated a signal on lane 1 but not 2 or 3 indicating it recognized the same epitope as N12, while N5 generated a signal on all 3 lanes indicating it recognized a different epitope than Abr 2.

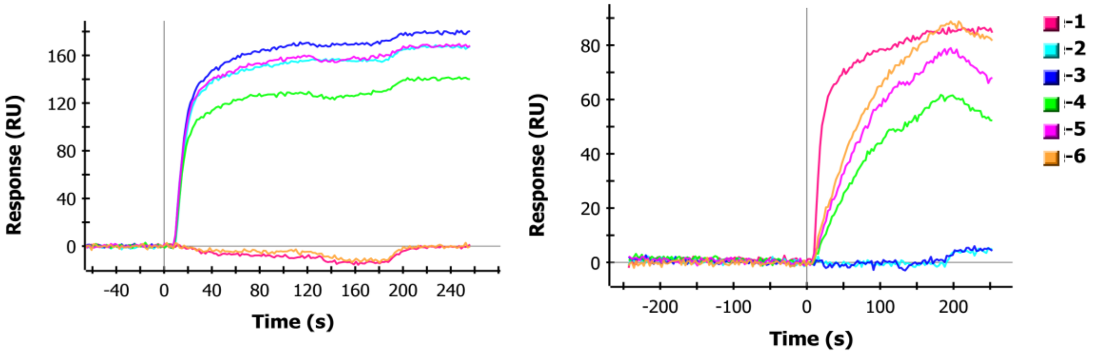

Figure S4. SPR data with fits for the binding of the selected sdAb to commercial abrin, abrax, *Abrus* agglutinin or abrin fractions. Time, in seconds, is on the X axis, while the Y axis if the SPR signal reported as resonance units (RU). Data and the calculated fits are shown. “No Binding” indicates that no binding was observed by SPR.

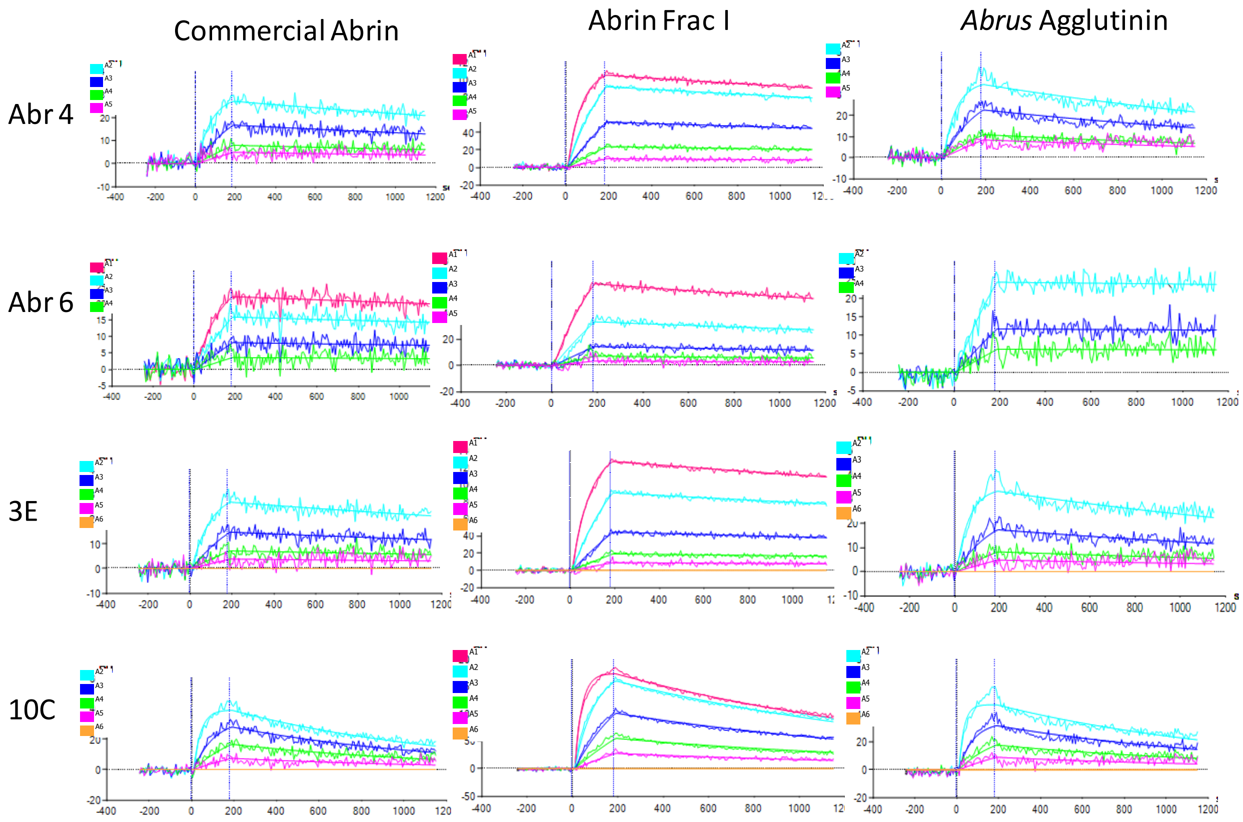
Commercial Abrin
Abrin Frac I
Abrus Agglutinin
Abr 4
Abr 6
3E
10C

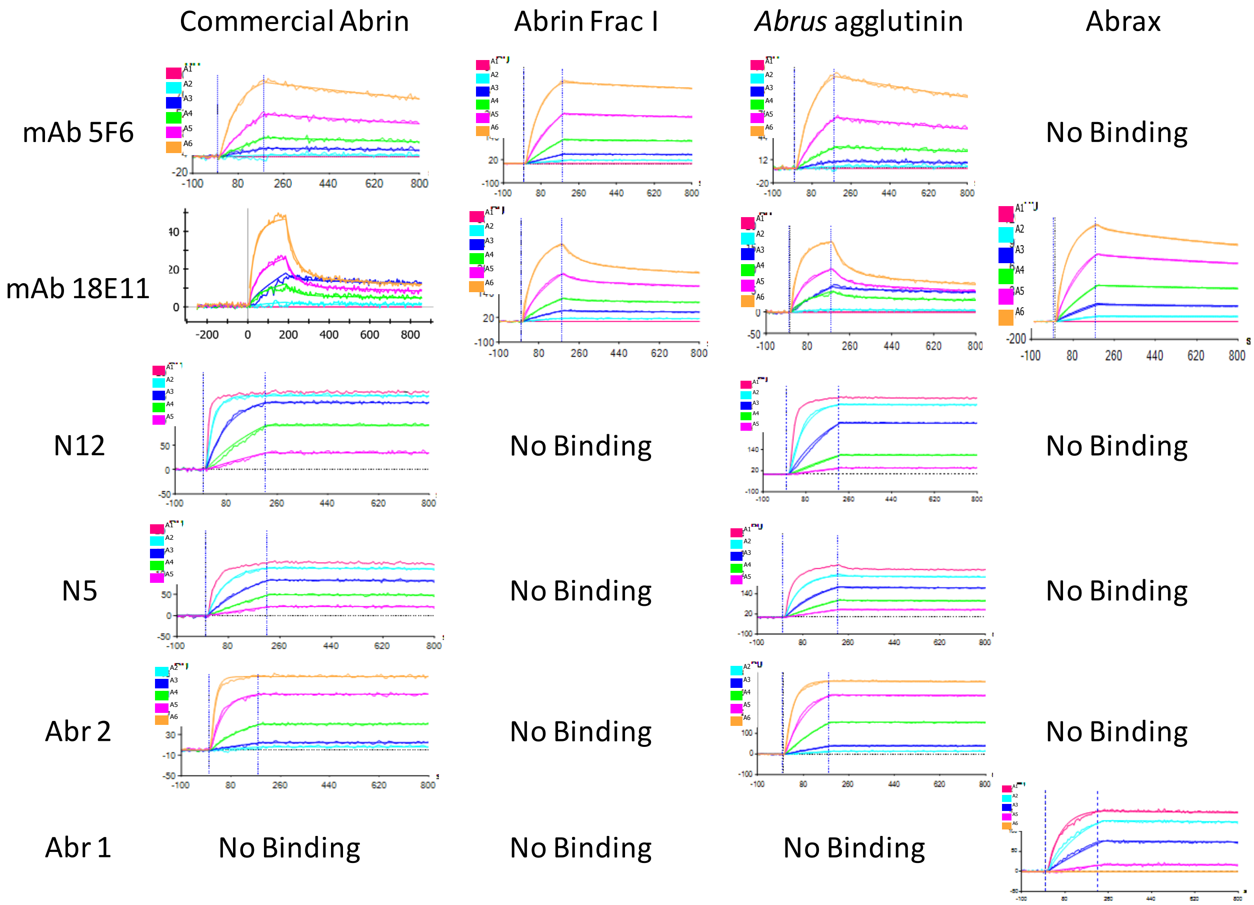
Commercial Abrin
Abrin Frac I
Abrus agglutinin
Abrax
mAb 5F6
No Binding
mAb 18E11
N12
No Binding
No Binding
N5
No Binding
No Binding
Abr 2
No Binding
No Binding
Abr 1
No Binding
No Binding
No Binding

Figure S5. Sandwich assays for the detection of commercial abrin (left), mixture of the abrin fractions (middle), and *Abrus* agglutinin (right) using sdAb and mAb captures paired with the indicated sdAb tracer. Captures are as follows: Orange triangle N12; blue square mAb 5F6; red circle and green diamond llama anti abrin; blue diamond mAb 30x anti-ricin. Concentration, in ng/mL, is on the X axis and signal, reported as median fluorescence intensity (MFI), is on the Y axis.

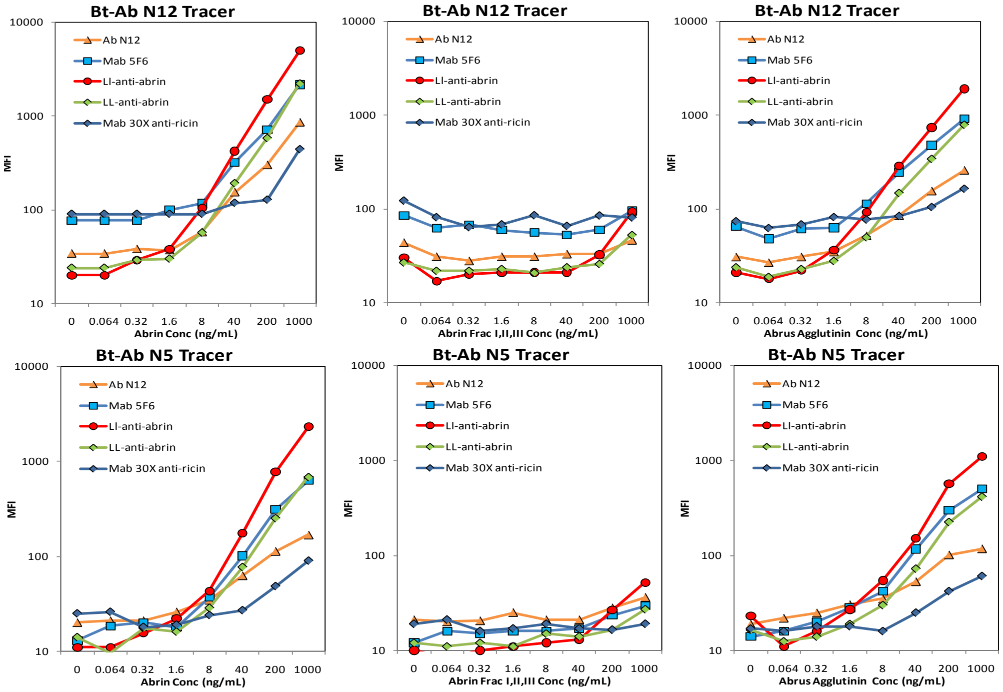

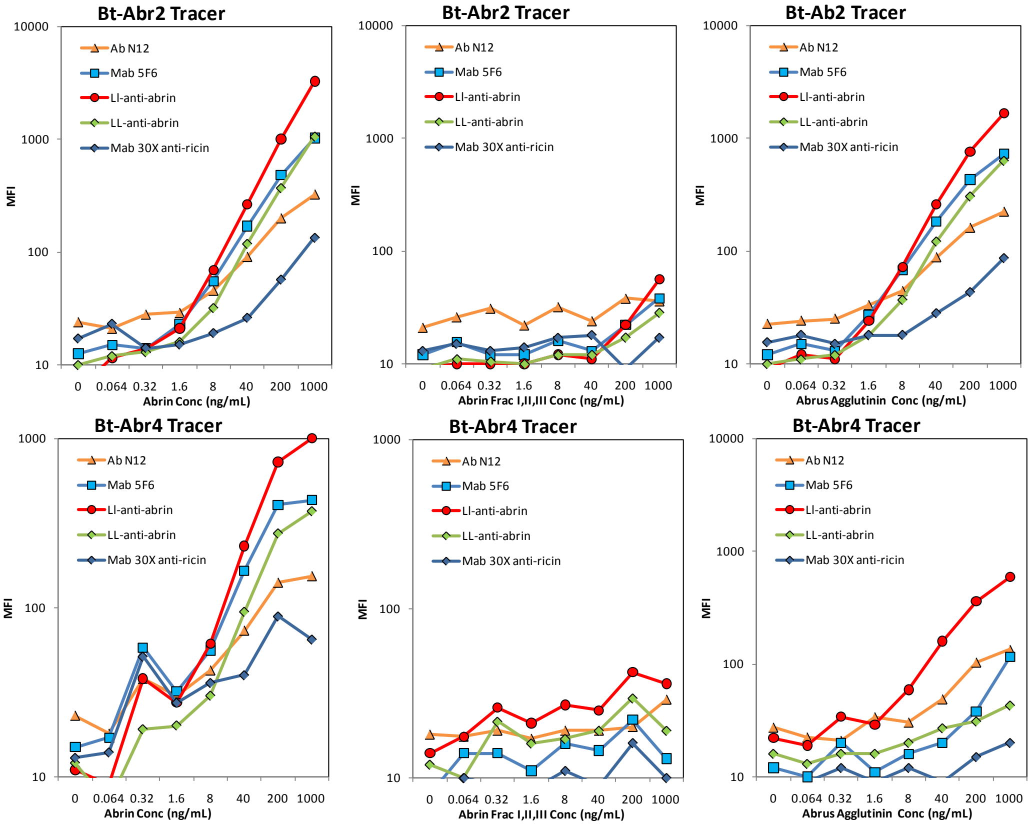

Figure S6. Native (top left) and denaturing (top right and bottom) gels. Labeled markers are shown on the left of each gel. In the top panels lanes 1–3 are abrin fractions I, II, III; lane 4 is *Abrus* Agglutinin; lane 5 is the commercial abrin; lane 6 is ricin and Lane 7 is RCA120. The bottom panel shows isolated recombinant abrin A chain (Abrax), analyzed by SDS-PAGE and Coomassie staining. The purified protein is 29 kDa consistent with the coding sequence of the abrin A chain.

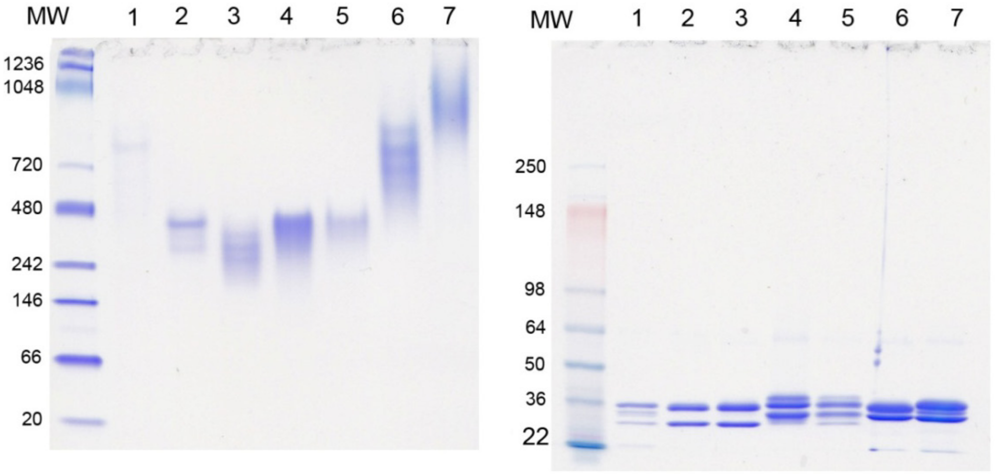

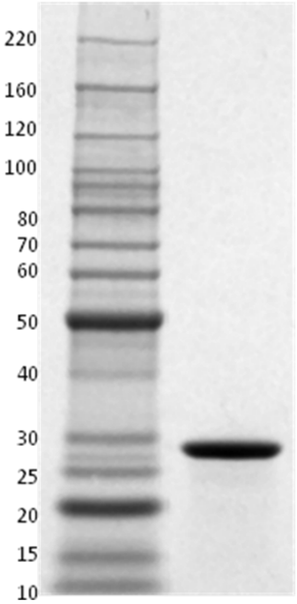
220
160
120
100
80
70
60
50
40
30
25
20
15
10
